# Rupture of the Uterus and Abdominal Parietes

**Published:** 1845-05

**Authors:** 

**Affiliations:** Director of the Obstetrical Institution of the city of Hildesheim


### SELECTIONS

FROM

### AMERICAN AND FOREIGN JOURNALS.

---

*Rupture of the Uterus and Abdominal Parietes.*—Cæsarean Operation performed with Success, both for the Mother and Infant. Thirteen months afterwards, Rupture of Uterus and Abdominal Parietes, from a second Pregnancy; Birth of the Infant by this spontaneous Opening, and perfect Recovery of the Mother. By Dr. Pearl, Director of the Obstetrical Institution of the city of Hildesheim.

A news vender, ætat, 28, enjoying good health, both during her early infancy and adult life, but deformed from rickets, in whom the catamenia appeared at the age of 13, entered the midwifery Hospital of Hildesheim, far advanced in pregnancy, shortly expecting to be brought to bed. A careful examination satisfactorily proved that the great diameter of the true pelvis, "du petit basin," was only two and a-half inches. It was, therefore, probable that she could only be delivered by the Cæsarean operation. Labour set in by powerful but useless pains, and the fœtus furnishing abundant proofs of life, the operation was determined on. Accordingly an incision was made in the direction of, and upon the linea alba; the peritoneum and the uterus were opened upon a director (sonde connelee,) and without the slightest lesion; a living female child, well formed, and arrived at full time, was drawn forth. By a slight traction upon the umbilical cord, the placenta was extracted through the same opening. The hemorrhage did not amount, at most, to a pound of blood. The edges of the uterine wound were then brought together as closely as possible by means of six "points" of suture, carefully avoiding the peritoneum. The lips of the tegumentary wound were placed in apposition, leaving, however, a small opening at the inferior angle, for the escape of any fluid that might otherwise accumulate within. During the operation, which was borne with the most heroic courage, the patient experienced only one slight nausea; she was not so much exhausted by it, and declared that she felt herself quite well. On the day following, the infant was applied to the breast, which it took with evident satisfaction. The issue of this accouchment was of the happiest nature, the mother suffering but a slight accession of fever, caused by an accumulation of milk; her infant refusing the breast, from being attacked with trismus nascentium, of which it died on the ninth day. The mother recovered perfectly, but slowly; at the expiration of two months from the operation there was complete cicatrization of the wound; at that of eight months, a reappearance of the catamenia; and in the ninth month, she left the hospital in excellent health, at which time the cicatrix was five inches in length and from half to three-quarters of an inch in width. In the beginning of the following year, she again became pregnant. During the first four months which followed, she enjoyed such good health, that she, for the first time noticed her state, by the movements of the infant in the twenty-first week. These movements were accompanied by violent dorsal pains, lasting but for a few minutes only; she paid but little attention to them, from having experienced similar at the age of sixteen. She now noticed an ulcerated spot upon the skin, which was slightly swollen; it was situated at the right side of the abdomen, at the distance of the width of the hand from the Cæsarean cicatrix. This ulceration daily extended in points, pouring out pus, and sometimes blood, causing but little or no pain; simple dressing was applied.

On the 14th of July, 1843, having taken cold, she had an access of fever, accompanied with pains in the sides, back, and abdomen, for which Dr. Shrœder was called in; he found that part of the abdominal integuments, which we have described, œdematous, and of a reddish-brown colour, and about the width of the hand in extent. The fœtus could be so distinctly felt through the abdominal parietes, that on first consideration it was considered to be extra-uterine. A solution of muriate of ammonia in liquorice juice was ordered, and the wound dressed with a mild ointment. The same evening, pains having the character of labour-pains, set in, but disappeared on the 16th. The muriate of ammonia was continued, causing a sensible diminution of the cough, and febrile excitement. In a short time, the patient was able to leave her bed; the movements of the fœtus were no more felt.

On the 18th, she experienced a pressing desire to make water, and go to stool; she arose, made a few steps forward, and called for her sister to hand her the vessel, when suddenly a crack (*craquement*) was heard. accompanied with a rupture of the abdominal parietes, through which the fœtus, enveloped in its membranes, could be felt. In this fearful dilemma, alone, and left to her own resources, with both hands, she retained the infant in the rupture. until help arrived. A midwife was called, who found the patient standing, the membranes being torn, she had merely time to take away the infant, which was dead. Dr. Shrœder, who arrived one hour after, found a large rent in the abdomen, situated below the umbilicus, of a transverse direction; the membranes, and a part of the omentum, protruded at the right, whilst the cord, and the placenta, still adhering to the uterus, appeared at the left. Fearing lest upon the extraction of the placenta, the intestines might escape, he called in Dr. Prael, on whose arrival, the patient, notwithstanding all she had suffered, was yet in a state not to be despaired of. She was pale, and the pulse was small; but there was not any thing in her manner, or the expression of her countenance, which indicated particular danger; the hemorrhage had not been very abundant, the wound smarted a little, but was not painful. The solution of continuity was at least five inches long, extending one and a half inches to the left side of the cicatrix, and three and a half inches to the right; its lips were so separated, that at first view, it was not easy to say whether it was transverse or perpendicular; its edges, particularly the inferior, were œdematous, swollen, turgid, turned inwards, and unequal; below this opening, and about an inch from its edge at right, there were two patches of extravasted blood, both transverse in direction, and one inch and a half in length. At the right of the centre of the rupture, a tumour, of a violet colour, and of the size of the head of a new born infant, was seen; it was the membranes, filled with coagulated blood: behind and above this, a portion of the omentum, as long as the hand, and the width of three fingers, protruded, the placenta in part visible, and a considerable portion of the cord filled the left side of the wound; there was no escape of the intestines. As the cord was so thin, that it could not bear the slightest traction, without danger of being torn, it was necessary to introduce three fingers into the uterus, through the abdominal opening, in order to remove the placenta. The uterus had already so much contracted, that it was quite impossible to learn, with certainty, the direction which the rupture had taken in it; the placenta, which was attached to the posterior wall of the uterus, gave trouble in its removal, and caused considerable pain to the patient.

During this operation, no part of the intestines protruded. In order to shun any such accident, the operator made the examination as short as possible, confining himself to washing away the blood, bringing the edges of the uterine opening together, and returning the omentum in the best way he could. The edges of the abdominal wound were so puckered (*plissès*), swollen, unequal, and œdematous, that sutures could not be used, they were therefore held together, by adhesive straps, placed perpendicularly. Having cut away with a scissors a small portion of the peritoneum, which was only held by a few filaments, at the right angle of the wound, the dressing was completed by soft compresses, and a bandage. An examination of the genital organs was next proceeded with, but nothing was found in the vagina. The orifice of the uterus was smooth; the os was dilated to about the width of two fingers, through which there was no flow of blood, or any other fluid. The reaction which followed so serious a lesion, was held within due limits, and there was but little fever. The state of the wound, however, gave rise to the most serious apprehension. At the removal of each dressing, a considerable quantity of sanious sanguineous fluid was discharged; its edges assumed an unhealthy aspect, and the patient complained of severe pains in the loins.

The opening, becoming gangrenous, gave issue to a very fœtid sanious discharge; however, with the exception of the stools, all the functions were normally performed, and the lochiat ransmitted through the natural passage. Quinine was administered, under the exhibition of which the aspect of the wound gradually changed, assuming a healthier appearance, and pus of a good quality was secreted. The quinine was now obliged to be discontinued from an attack of rheumatism of the left side, but was afterwards administered with good effect, notwithstanding an abscess formed in the left inguinal region, carrying in its train a swelling, and paralysis of the leg of that side. Emollient poultices were applied, but the œdematous swelling progressed, extending over the entire abdominal integuments. The process of cicatrization, which had reduced the wound to the size of a two-franc piece, was also manifestly arrested. By the aid of tonics, antiseptics, and a nutritious regimen, the œdema disappeared in the course of eight days; sleep and appetite now returned, her strength augmented, cicatrization recommenced, the quantity of pus daily diminished, and there only remained a few cutaneous ulcerations in the neighborhood of the rupture, which were soon healed by the application of the nit. argenti. In one month after she was so far re-established in health as to be able to quit her bed; and in fifteen days more, the wound was reduced to the diameter of a one-franc piece.

She was now enabled to be engaged in domestic affairs, and walk for half an hour each day. On the 5th of October, cicatrization was complete, she felt no pain, and was fast regaining her *embonpoint*. The catamenia appeared in six weeks after the accident, and again towards the end of October without pain, but less abundant than the first time. She now left the hospital.

This extraordinary case is so much the more interesting, as it is perhaps the only one in the annals of medical science. One exhibiting some analogy is related in an old English work, little known, published by the Society of Edinburgh.

The translator, not without some difficulty, from the indefinite description given of this work, found the paper referred to in vol. ii. of the "New Edinburgh Essays," p. 338, published in 1756. It was read before the Society by Alexander Monro, senior, and is thus given:

"In April, 1736, Elspet Grant, in the parish of Moy, being with child, took her labour pains. After they had continued three days with the child in birth, two cracks, as if the rafters of the house had broken, were heard about the sick wife, and her belly was rent from near the navel, with a squaint downwards, and to the left side to near the share bone. At this rent the child came into the world, the after-burden was brought away, and the entrails were seen. The woman recovered."—*Dub. Jour.*

